# A mixed methods case study exploring the impact of membership of a multi-activity, multicentre community group on social wellbeing of older adults

**DOI:** 10.1186/s12877-018-0913-1

**Published:** 2018-09-24

**Authors:** Gabrielle Lindsay-Smith, Grant O’Sullivan, Rochelle Eime, Jack Harvey, Jannique G. Z. van Uffelen

**Affiliations:** 1 Institute for Health and Sport, Victoria University, Melbourne, Australia; 2 School of Health and Life Sciences, Federation University, Ballarat, Australia; 3 Department of Movement Sciences, Physical Activity, Sports and Health Research Group, KU Leuven - University of Leuven, Leuven, Belgium

**Keywords:** Ageing, Social support, Social engagement, Friendship, Loneliness, Retirement, Group activity

## Abstract

**Background:**

Social wellbeing factors such as loneliness and social support have a major impact on the health of older adults and can contribute to physical and mental wellbeing. However, with increasing age, social contacts and social support typically decrease and levels of loneliness increase. Group social engagement appears to have additional benefits for the health of older adults compared to socialising individually with friends and family, but further research is required to confirm whether group activities can be beneficial for the social wellbeing of older adults.

**Methods:**

This one-year longitudinal mixed methods study investigated the effect of joining a community group, offering a range of social and physical activities, on social wellbeing of adults with a mean age of 70. The study combined a quantitative survey assessing loneliness and social support (*n* = 28; three time-points, analysed using linear mixed models) and a qualitative focus group study (*n* = 11, analysed using thematic analysis) of members from Life Activities Clubs Victoria, Australia.

**Results:**

There was a significant reduction in loneliness (*p* = 0.023) and a trend toward an increase in social support (*p* = 0.056) in the first year after joining. The focus group confirmed these observations and suggested that social support may take longer than 1 year to develop. Focus groups also identified that group membership provided important opportunities for developing new and diverse social connections through shared interest and experience. These connections were key in improving the social wellbeing of members, especially in their sense of feeling supported or connected and less lonely. Participants agreed that increasing connections was especially beneficial following significant life events such as retirement, moving to a new house or partners becoming unwell.

**Conclusions:**

Becoming a member of a community group offering social and physical activities may improve social wellbeing in older adults, especially following significant life events such as retirement or moving-house, where social network changes. These results indicate that ageing policy and strategies would benefit from encouraging long-term participation in social groups to assist in adapting to changes that occur in later life and optimise healthy ageing.

## Background

### Ageing population and the need to age well

Between 2015 and 2050 it is predicted that globally the number of adults over the age of 60 will more than double [1]. Increasing age is associated with a greater risk of chronic illnesses such as cardio vascular disease and cancer [2] and reduced functional capacity [3, 4]. Consequently, an ageing population will continue to place considerable pressure on the health care systems.

However, it is also important to consider the individuals themselves and self-perceived good health is very important for the individual wellbeing and life-satisfaction of older adults [5]. The terms “successful ageing” [6] and “healthy ageing” [5] have been used to define a broader concept of ageing well, which not only includes factors relating to medically defined health but also wellbeing. Unfortunately, there is no agreed definition for what exactly constitutes healthy or successful ageing, with studies using a range of definitions. A review of 28 quantitative studies found that successful ageing was defined differently in each, with the majority only considering measures of disability or physical functioning. Social and wellbeing factors were included in only a few of the studies [7].

In contrast, qualitative studies of older adults’ opinions on successful ageing have found that while good physical and mental health and maintaining physical activity levels are agreed to assist successful ageing, being independent or doing something of value, acceptance of ageing, life satisfaction, social connectedness or keeping socially active were of greater importance [8–10].

In light of these findings, the definition that is most inclusive is “healthy ageing” defined by the World Health Organisation as “the process of developing and maintaining the functional ability (defined as a combination of intrinsic capacity and physical and social environmental characteristics), that enables well-being in older age” (p28) [5].This definition, and those provided in the research of older adults’ perceptions of successful ageing, highlight social engagement and social support as important factors contributing to successful ageing, in addition to being important social determinants of health [11, 12].

Social determinants of health, including loneliness and social support, are important predictors of physical, cognitive and mental health and wellbeing in adults [12] and older adults [13–15]. Loneliness is defined as a perception of an inadequacy in the quality or quantity of one’s social relationships [16]. Social support, has various definitions but generally it relates to social relationships that are reciprocal, accessible and reliable and provide any or a combination of supportive resources (e.g. emotional, information, practical) and can be measured as perceived or received support [17]. These types of social determinants differ from those related to inequality (health gap social determinants) and are sometimes referred to as ‘social cure’ social determinants [11]. They will be referred to as ‘social wellbeing’ outcome measures in this study.

Unfortunately, with advancing age, there is often diminishing social support, leading to social isolation and loneliness [18, 19]. Large nationally representative studies of adults and older adults reported that social activity predicted maintenance or improvement of life satisfaction as well as physical activity levels [20], however older adults spent less time in social activity than middle age adults.

### Social wellbeing and health

A number of longitudinal studies have found that social isolation for older adults is a significant predictor of mortality and institutionalisation [21–23]. A meta-analysis by Holt-Lunstadt [12] reported that social determinants of health, including social integration and social support (including loneliness and lack of perceived social support) to be equal to, or a greater risk to mortality as common behavioural risk factors such as smoking, physical inactivity and obesity. Loneliness is independently associated with poor physical and mental health in the general population, and especially in older adults [13–15]. Adequate perceived social support has also been consistently associated with improved mental and physical health in both general and older adults [20, 24–29]. The mechanism suggested for this association is that social support buffers the negative impacts of stressful situations and life events [30]. The above research demonstrates the benefit of social engagement for older adults; in turn this highlights the importance of strategies that reduce loneliness and improve social support and social connectedness for older adults.

Socialising in groups seems to be especially important for the health and wellbeing of older adults who may be adjusting to significant life events [26, 31–33]. This is sometimes referred to as social engagement or social companionship [26, 30, 31]. It seems that the mechanism enabling such health benefits with group participation is through strengthening of social identification, which in turn increases social support [31, 34, 35]. Furthermore, involvement in community groups can be a sustainable strategy to reduce loneliness and increase social support in older adults, as they are generally low cost and run by volunteers [36–39].

Despite the demonstrated importance of social factors for successful ageing and the established risk associated with reduced social engagement as people age, few in-depth studies have longitudinally investigated the impact of community groups on social wellbeing. For example, a non-significant increase in social support and reduction in depression was found in a year-long randomised controlled trial conducted in senior centres in Norway with lonely older adults in poor physical and mental health [37]. Some qualitative studies have reported that community groups and senior centres can contribute to fun and socialisation for older adults, however social wellbeing was not the primary focus of the studies [38, 40, 41]. Given that social wellbeing is a broad and important area for the health and quality of life in older adults, an in-depth study is warranted to understand how it can be maximised in older adults. This mixed methods case study of an existing community aims to: i) examine whether loneliness and social support of new members of Life Activities Clubs (LACs) changes in the year after joining and ii) conduct an in-depth exploration of how social wellbeing changes in new and longer-term members of LACs.

## Methods

### Design

A mixed methods study was chosen as the design for this research to enable an in-depth exploration of how loneliness and social support may change as a result of joining a community group. A case study was conducted using a concurrent mixed-methods design, with a qualitative component giving context to the quantitative results. Where the survey focused on the impact of group membership on social support and loneliness, the focus groups were an open discussion of the benefits in the lived context of LAC membership. The synthesis of the two sections of the study was undertaken at the time of interpretation of the results [42].

The two parts of our study were as follows:

- a longitudinal survey (three time points over 1 year: baseline, 6 and 12 months). This part of the study formed the quantitative results;
- a focus group study of members of the same organisation (qualitative).

Ethics approval to conduct this study was obtained from the Victoria University Human Research Ethics Committee (HRE14–071 [survey] and HRE15–291 [focus groups]) All participants provided informed consent to partake in the study prior to undertaking the first survey or focus group.

### Setting and participants

#### Life activities clubs Victoria

Life Activities Clubs Victoria (LACVI) is a large not-for-profit group with 23 independently run Life Activities Clubs (LACs) based in both rural and metropolitan Victoria. It has approximately 4000 members. The organisation was established to assist in providing physical, social and recreational activities as well as education and motivational support to older adults managing significant change in their lives, especially retirement.

#### Survey

Eighteen out of 23 LAC clubs agreed to take part in the survey study. During the sampling period from May 2014 to December 2016, new members from the participating clubs were given information about the study and invited to take part. Invitations took place in the form of flyers distributed with new membership material.

##### Inclusion/ exclusion criteria

Community-dwelling older adults who self-reported that they could walk at least 100 m and who were new members to LACVI and able to complete a survey in English were eligible to participate. New members were defined as people who had never been members of LACVI or who had not been members in the last 2 years.

To ensure that the cohort of participants were of a similar functional level, people with significant health problems limiting them from being able to walk 100 m were excluded from participating in the study.

Once informed consent was received, the participants were invited to complete a self-report survey in either paper or online format (depending on preference). This first survey comprised the baseline data and the same survey was completed 6 months and 12 months after this initial time point. Participants were sent reminders if they had not completed each survey more than 2 weeks after each was delivered and then again 1 week later.

#### Focus groups

Two focus groups (FGs) were conducted with new and longer-term members of LACs. The first FG (*n* = 6) consisted of members who undertook physical activity in their LAC (e.g. walking groups, tennis, cycling). The second FG (*n* = 5) consisted of members who took part in activities with a non-physical activity (PA) focus (e.g. book groups, social groups, craft or cultural groups). LACs offer both social and physical activities and it was important to the study to capture both types of groups, but they were kept separate to assist participants in feeling a sense of commonality with other members and improving group dynamic and participation in the discussions [43]. Of the people who participated in the longitudinal survey study, seven also participated in the FGs.

The FG interviews were facilitated by one researcher (GLS) and notes around non-verbal communication, moments of divergence and convergence amongst group members, and other notable items were taken by a second researcher (GOS). Both researchers wrote additional notes after the focus groups and these were used in the analysis of themes. Focus groups were recorded and later transcribed verbatim by a professional transcriptionist, including identification of each participant speaking. One researcher (GLS) reviewed each transcription to check for any errors and made any required modifications before importing the transcriptions into NVivo for analysis. The transcriber identified each focus group participant so themes for individuals or other age or gender specific trends could be identified.

### Dependent variables

#### Social support

Social support was assessed using the Duke–UNC Functional Social support questionnaire [44]. This scale specifically measures participant perceived functional social support in two areas; i) confidant support (5 questions; e.g. chances to talk to others) and ii) affective support (3 questions; e.g. people who care about them). Participants rated each component of support on a 5-item likert scale between *‘much less than I would like’* (1 point) to *‘as much as I would like’* (5 points). The total score used for analysis was the mean of the eight scores (low social support = 1, maximum social support = 5). Construct validity, concurrent validity and discriminant validity are acceptable for confidant and affective support items in the survey in the general population [44].

#### Loneliness

Loneliness was measured using the de Jong Gierveld and UCLA-3 item loneliness scales developed for use in many populations including older adults [45]. The 11-item de Jong Gierveld loneliness scale (DJG loneliness) [46] is a multi-dimensional measure of loneliness and contains five positively worded and six negatively worded items. The items fall into four subscales; feelings of severe loneliness, feelings connected with specific problem situations, missing companionship, feelings of belongingness. The total score is the sum of the items scores (i.e. 11–55): 11 is low loneliness and 55 is severe loneliness. Self-administered versions of this scale have good internal consistency (> = 0.8) and inter-item homogeneity and person scalability that is as good or better than when conducted as face-to face interviews. The validity and reliability for the scale is adequate [47]. The UCLA 3-item loneliness scale consists of three questions about how often participants feel they lack companionship, feel left out and feel isolated. The responses are given on a three-point scale ranging from hardly ever (1) to often (3). The final score is the sum of these three items with the range being from lowest loneliness (3) to highest loneliness (9). Reliability of the scale is good, (alpha = 0.72) as are discriminant validity and internal consistency [48]. The scale is commonly used to measure loneliness with older adults ([49] – review), [50, 51].

#### Sociodemographic variables

The following sociodemographic characteristics were collected in both the survey and the focus groups: age, sex, highest level of education, main life occupation [52], current employment, ability to manage on income available, present marital status, country of birth, area of residence [53]. They are categorised as indicated in Table 2.

#### Health variables

The following health variables were collected: *Self-rated general health (from SF-12)* [54] and *Functional health (ability to walk 100 m- formed part of the inclusion criteria)* [55]. See Table 2 for details about the categories of these variables.

### Analysis

#### Survey

The effects of becoming a member on quantitative outcome variables (i.e. Social support, DJG loneliness and UCLA loneliness) were analysed using linear mixed models (LMM). LMM enabled testing for the presence of intra-subject random effects, or equivalently, correlation of subjects’ measures over time (baseline, 6-months and 12 months). Three correlation structures were examined: independence (no correlation), compound symmetry (constant correlation of each subjects’ measures over the three time points) and autoregressive (correlation diminishing with increase in spacing in time). The best fitting correlation structure was compound symmetry; this is equivalent to a random intercept component for each subject. The LMM incorporated longitudinal trends over time, with adjustment for age as a potential confounder. Statistical analyses were conducted using SPSS for windows (v24).

UCLA loneliness and social support residuals were not normally distributed and these scales were Log10 transformed for statistical analysis.

Analyses were all adjusted for age, group attendance (calculated as average attendance at 6 and 12 months) and employment status at baseline (Full-time, Part-time, not working).

#### Focus groups

Focus group transcripts were analysed using thematic analysis [56, 57], a flexible qualitative methodology that can be used with a variety of epistemologies, approaches and analysis methods [56]. The transcribed data were analysed using a combination of theoretical and inductive thematic analysis [56]. It was theorised that membership in a LAC would assist with social factors relating to healthy ageing [5], possibly through a social identity pathway [58], although we wanted to explore this. Semantic themes were drawn from these codes in order to conduct a pragmatic evaluation of the LACVI programs [56]. Analytic rigour in the qualitative analysis was ensured through source and analyst triangulation. Transcriptions were compared to notes taken during the focus groups by the researchers (GOS and GLS). In addition, Initial coding and themes (by GLS) were checked by a second researcher (GOS) and any disagreements regarding coding and themes were discussed prior to finalisation of codes and themes [57].

## Results

### Survey

Sociodemographic and health characteristics of the 28 participants who completed the survey study are reported in Table 1. The mean age of the participants was 66.9 and 75% were female. These demographics are representative of the entire LACVI membership. Education levels varied, with 21% being university educated, and the remainder completing high school or technical certificates. Two thirds of participants were not married. Some sociodemographic characteristics changed slightly at 6 and 12 months, mainly employment (18% in paid employment at baseline and 11% at 12-months) and ability to manage on income (36% reporting trouble managing on their income at baseline and 46% at 12 months). Almost 90% of the participants described themselves as being in good-excellent health.

**Table 1:** Sociodemographic and health characteristics of survey and focus group respondents at baseline

Sociodemographic characteristics		Survey respondents (*n* = 28)	Focus groups (*n* = 11)
Age in years, mean (SD)		66.9 (9.0)	67.1 (5.9)
Sex, n (%)	Male	7 (25)	2 (18)
	Female	21 (75)	9 (82)
Highest level of education, n (%)	Completed primary school	0 (0)	1 (9)
	Up to year 12	10 (36)	3 (27)
	Technical studies/ trade certificate	10 (36)	4 (36)
	Tertiary studies	6 (21)	3 (27)
	Missing	2 (7)	0
Main life occupation, n (%)	Manager	4 (14)	2 (18)
	Professional	10 (36)	4 (3)
	Clerical	9 (32)	5 (45)
	Trade, production or labour	5 (18)	0
Current employment, n (%)	Full-time	2 (7)	0
	Part-time/casual	3 (11)	2 (18)
	Not in paid employment	23 (82)	9 (81)
Ability to manage on Income, n (%)	Very difficult	2 (7)	0
	Somewhat difficult	8 (29)	3 (27)
	Not difficult	18 (64)	8 (18)
Present marital status, n (%)	Not married	17 (61)	8 (73)
	Married/defacto	11 (40)	3 (27)
Country of birth, n (%)	Australia	23 (82)	8 (73)
	Other	5 (18)	3 (27)
Area of residence, n (%)	Urban	23 (82)	9 (82)
	Rural	5 (18)	2 (18)
Health			
General health, n (%)	Very good- excellent	16 (57)	NA
	Good	9 (32)	NA
	Fair	3 (11)	NA
Functional health (Walking limitation), n (%)	Some limitation	2 (7)	NA
	No limitation	26 (93)	NA

### Types of activities

There were a variety of types of activities that participants took part in: physical activities such as walking groups (*n* = 7), table tennis (*n* = 5), dancing class (*n* = 2), exercise class (*n* = 1), bowls (*n* = 2), golf (*n* = 3), cycling groups (*n* = 1) and non-physical leisure activities such as art and literature groups (*n* = 5), craft groups (*n* = 5), entertainment groups (*n* = 12), food/dine out groups (*n* = 18) and other sedentary leisure activities (e.g. mah jong, cards),(*n* = 4). A number of people took part in more than one activity.

#### Frequency of attendance at LACVI and changes in social wellbeing

At six and 12 months, participants indicated how many times in the last month they attended different types of activities at their LAC. Most participants maintained the same frequency of participation over both time points. Only four people participated more frequently at 12 than at 6 months and nine reduced participation levels. The latter group included predominantly those who reduced from more than two times per week at 6 months to 2×/week at 6 months to one to two times per week (*n* = 5) or less than one time per week (*n* = 2) at 12 months. Average weekly club attendance at six and 12 months was included as a covariate in the statistical model.

#### Outcome measures

**Fig. 1:**
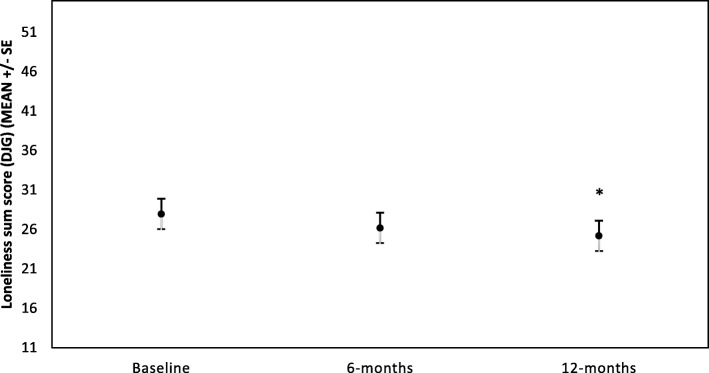
DJG loneliness for all participants over first year of membership at LAC club (*n* = 28). *Represents significant difference compared to baseline (*p* < 0.01)

**Fig. 2:**
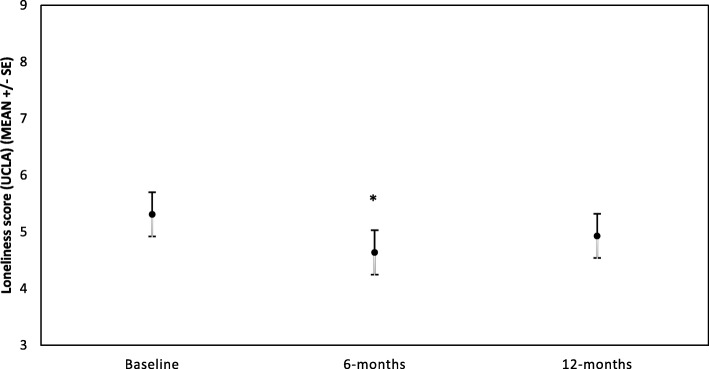
UCLA loneliness score for all participants over first year of membership at LAC club (*n* = 28). *Indicates log values of the variable at 6-months were significantly different from baseline (*p* < 0.01)

Overall, participants reported moderate social support and loneliness levels at baseline (See Table 2). Loneliness, as measured by both scales, reduced significantly over time. There was a significant effect of time on the DJG loneliness scores (F (2, 52) = 3.83, *p* = 0.028), with Post-Hoc analysis indicating a reduction in DJG loneliness between baseline and 12 months (*p* = 0.008). UCLA loneliness scores (transformed variable) also changed significantly over time (F (2, 52) = 4.08, *p* = 0.023). Post hoc tests indicated a reduction in UCLA loneliness between baseline and 6 months (*p* = 0.007). There was a small non-significant increase in social support (F (2, 53) =2.88, *p* = 0.065) during the first year of membership (see Table 2 and Figs. 1 and 2).

**Table 2:** Means and standard errors for social wellbeing variables over time

Variable	Baseline (*n* = 28)	6 months (*n* = 27)	12 months (*n* = 28)	*p*-value
Duke social support^a^	3.83 (0.2)	4.08 (0.2)	4.09 (0.2)	0.065^d^
DJG loneliness^b^	27.95 (1.94)	26.18 (1.94)	25.17 (1.94)	0.028*
UCLA loneliness^c^	5.31 (0.39)	4.64 (0.39)	4.93 (0.39)	0.023*^d^

*significant effect of time for the indicated variable at *p* < 0.05

All analyses are adjusted for age, employment at baseline, and mean weekly LAC attendance

^a^Duke_UNC functional social support scale. Range 1–5. High social support = 5. *p*-value represents the *p*-value for the log-transformed variable

^b^De Jong Gierveld loneliness scale. Scored as a 5 item likert scale from Yes!, yes, more or less, no, No! Range = 11–55. Highest loneliness = 55.

^c^UCLA 3-item loneliness scale. Range = 3–9. Highest loneliness = 9

^d^*p*-value presented here are log-transformed variable analyses

#### Focus groups

In total, 11 participants attended the two focus groups, six people who participated in PA clubs (four women) and five who participated in social clubs (all women). All focus group participants were either retired (*n* = 9) or semi-retired (*n* = 2). The mean age of participants was 67 years (see Table 2 for further details). Most of the participants (82%) had been members of a LAC for less than 2 years and two females in the social group had been members of LAC clubs for 5 and 10 years respectively.

**Fig. 3:**
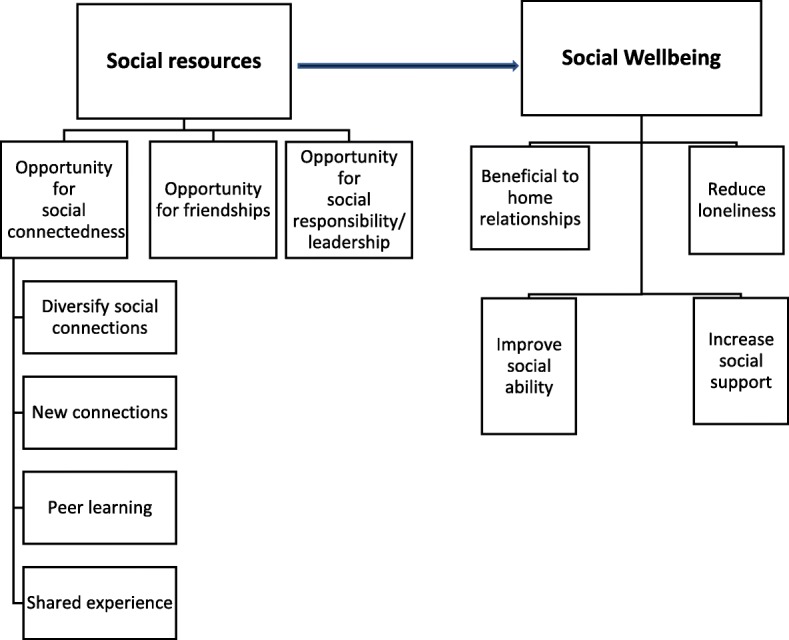
Themes arising from focus group discussion around the benefits of LAC membership

Analysis of the focus group transcripts identified two themes relating to social benefits of group participation; i) Social resources and ii) Social wellbeing (see Fig. 3). Group discussion suggested that membership of a LAC provides access to more social resources through greater and diverse social contact and opportunity. It is through this improvement in social resources that social wellbeing may improve.

### Social resources

The social resources theme referred to an increase in the availability and variety of social connections that resulted from becoming a member of a LAC. The social nature of the groups enabled an expansion and diversification of members’ social network and improved their sense of social connectedness. There was widespread agreement in both the focus groups that significant life events, especially retirement, illness or death of spouse and moving house changes one’s social resources. Membership of the LAC had benefits especially at these times and these events were often motivators to join such a club. Most participants found that their social resources declined after retirement and even felt that they were grieving for the loss of their work.

“*I just saw work as a collection of, um, colleagues as opposed to friends. I had a few good friends there. Most were simply colleagues or acquaintances …. [interviewer- Mmm.] ..Okay, you’d talk to them every day. You’d chatter in the kitchen, oh, pass banter back and forth when things are busy or quiet, but... Um, in terms of a friendship with those people, like going to their home, getting to know them, doing other things with them, very few. But what I did miss was the interaction with other people. It had simply gone….. But, yeah, look, that, the, yeah, that intervening period was, oh, a couple of months. That was a bit tough…. But in that time the people in LAC and the people in U3A…. And the other dance group just drew me into more things. Got to know more people. So once again, yeah, reasonable group of acquaintances.”* (Male, PAFG)

Group members indicated general agreement with these two responses, however one female found she had a greater social life following retirement due to the busy nature of her job.

Within the social resources theme, three subthemes were identified, *i) Opportunity for social connectedness, ii) Opportunity for friendships, and iii) Opportunity for social responsibility/leadership*. Interestingly, these subthemes were additional to the information gathered in the survey. This emphasises the power of the inductive nature of the qualitative exploration employed in the focus groups to broaden the knowledge in this area.

The most discussed and expanded subtheme in both focus groups was *Opportunity for social connectedness*, which arose through developing new connections, diversifying social connections, sharing interests and experiences with others and peer learning. Participants in both focus groups stated that being a member of LAC facilitated their socialising and connecting with others to share ideas, skills and to do activities with, which was especially important through times of significant life events. Furthermore, participants in each of the focus groups valued developing diverse connections:

“*Yeah, I think, as I said, I finished up work and I, and I had more time for wa-, walking. So I think a, in meeting, in going to this group which, I saw this group of women but then someone introduced me to them. They were just meeting, just meeting a new different set of people, you know? As I said, my work people and these were just a whole different group of women, mainly women. There’s not many men. [Interviewer: Yes.]….. Although our leader is a man, which is ironic and is about, this man out in front and there’s about 20 women behind him, but, um, so yeah, and people from different walks of life and different nationalities there which I never knew in my work life, so yeah. That’s been great. So from that goes on other things, you know, you might, uh, other activities and, yeah, people for coffee and go to the pictures or something, yeah. That’s great.” (Female, PAFG)*

Simply making new connections was the most widely discussed aspect related to the opportunity for social connectedness subtheme, with all participants agreeing that this was an important benefit of participation in LAC groups.

*“Well, my experience is very similar to everybody else’s…….: I, I went from having no social life to a social life once I joined a group.” (Female, PAFG)*

There was agreement in both focus groups that these initial new connections made at a LAC are strengthened through development of deeper personal connections with others who have similar demographics and who are interested in the same activities. This concurs with the Social Identity Theory [58] discussed previously.

*“and I was walking around the lake in Ballarat, like wandering on my own. I thought, This is ridiculous. I mean, you’ve met all those groups of women coming the opposite way, so I found out what it was all about, so I joined, yeah. So that’s how I got into that.[ Interviewer: Yeah.] Basically sick of walking round the lake on my own. [Interviewer: Yeah, yeah.] So that’s great. It’s very social and they have coffee afterwards which is good.”* (female, PAFG)

The subtheme *Opportunity for development of friendships* describes how, for some people, a number of LAC members have progressed from being just initial social connections to an established friendship. This signifies the strength of the connections that may potentially develop through LAC membership. Some participants from each group mentioned friendships developing, with slightly more discussion of this seen in the social group.

*“we all have a good old chat, you know, and, and it’s all about friendship as well.” (female, SocialFG)*

The subtheme *Opportunity for social responsibility or leadership* was mentioned by two people in the active group, however it was not brought up in the social group. This opportunity for leadership is linked with the development of a group identity and desiring to contribute meaningfully to a valued group.

*“with our riding group, um, you, a leader for probably two rides a year so you’ve gotta prepare for it, so some of them do reccie rides themselves, so, um, and also every, uh, so that’s something that’s, uh, a responsibility.” (male, PAFG)*

### Social wellbeing

The social resources described above seem to contribute to a number of social, wellbeing outcomes for participants. The sub themes identified for Social wellbeing were*, i) Increased social support, ii) Reduced loneliness, iii) Improved home relationships* and *iv) Improved social skills.*

#### Increased social support

Social support was measured quantitatively in the survey (no significant change over time for new members) and identified as a benefit of LAC membership during the focus group discussions. However, only one of the members of the active group mentioned social support directly.

*‘it’s nice to be able to pick up the phone and share your problem with somebody else, and that’s come about through LAC. ……‘Cos before that it was through, with my family (female, PAFG)*

There was some agreement amongst participants of the PA group that they felt this kind of support may develop in time but most of them had been members for less than 2 years.

*“[Interviewer: Yeah. Does anyone else have that experience?* (relating to above quote)]” *There is one lady but she’s actually the one that I joined with anyway. [Interviewer: Okay.] But I, I feel there are others that are definitely getting towards that stage. It’s still going quite early days. (female1, PAFG) [Interviewer: I guess it’s quite early for some of you, yeah.] “yeah” (female 2, PAFG)*

Social support through sharing of skills was mentioned by one participant in the social group also, with agreement indicated by most of the others in the social focus group.

Discussion in the focus groups also touched on the subthemes *Reduced loneliness* and *Improved home relationships,* which were each mentioned by one person. And focus groups also felt that group membership *Improved social skills* through opening up and becoming more approachable (male, PAFG) or enabling them to become more accepting of others’ who are different (general agreement in Social FG).

## Discussion

This case study integrated results from a one-year longitudinal survey study and focus group discussions to gather rich information regarding the potential changes in social wellbeing that older adults may experience when joining community organisations offering group activities. The findings from this study indicate that becoming a member of such a community organisation can be associated with a range of social benefits for older adults, particularly related to reducing loneliness and maintaining social connections.

### Loneliness

Joining a LAC was associated with a reduction in loneliness over 1 year. This finding is in line with past group-intervention studies where social activity groups were found to assist in reducing loneliness and social isolation [49]. This systematic review highlighted that the majority of the literature explored the effectiveness of group activity interventions for reducing severe loneliness or loneliness in clinical populations [49]. The present study extends this research to the general older adult population who are not specifically lonely and reported to be of good general health, rather than a clinical focus. Our findings are in contrast to results from an evaluation of a community capacity-building program aimed at reducing social isolation in older adults in rural Australia [59]. That program did not successfully reduce loneliness or improve social support. The lack of change from pre- to post-program in that study was reasoned to be due to sampling error, unstandardised data collection, and changes in sample characteristics across the programs [59]. Qualitative assessment of the same program [59] did however suggest that participants felt it was successful in reducing social isolation, which does support our findings.

Changes in loneliness were not a main discussion point of the qualitative component of the current study, however some participants did express that they felt less lonely since joining LACVI and all felt they had become more connected with others. This is not so much of a contrast in results as a potential situational issue. The lack of discussion of loneliness may have been linked to the common social stigma around experiencing loneliness outside certain accepted circumstances (e.g. widowhood), which may lead to underreporting in front of others [45].

Overall, both components of the study suggest that becoming a member of an activity group may be associated with reductions in loneliness, or at least a greater sense of social connectedness. In addition to the social nature of the groups and increased opportunity for social connections, another possible link between group activity and reduced loneliness is an increased opportunity for time out of home. Previous research has found that more time away from home in an average day is associated with lower loneliness in older adults [60]. Given the significant health and social problems that are related to loneliness and social isolation [13–15], the importance of group involvement for newly retired adults to prevent loneliness should be advocated.

### Social support

In line with a significant reduction in loneliness, there was also a trend (*p* = 0.056) toward an increase in social support from baseline to 12 months in the survey study. Whilst suggestive of a change, it is far less conclusive than the findings for loneliness. There are a number of possible explanations for the lack of statistically significant change in this variable over the course of the study. The first is the small sample size, which would reduce the statistical power of the study. It may be that larger studies are required to observe changes in social support, which are possibly only subtle over the course of 1 year. This idea is supported by a year-long randomised controlled trial with 90 mildly-depressed older adults who attended senior citizen’s club in Norway [37]. The study failed to see any change in general social support in the intervention group compared to the control over 1 year. Additional analysis in that study suggested that people who attended the intervention groups more often, tended to have greater increases in SS (*p* = 0.08). The researchers stated that the study suffered from significant drop-out rates and low power as a result. In this way, it was similar to our findings and suggests that social support studies require larger numbers than we were able to gain in this early exploratory study. Another possible reason for small changes in SS in the current study may be the type of SS measured. The scale used gathered information around functional support or support given to individuals in times of need. Maybe it is not this type of support that changes in such groups but more specific support such as task-specific support. It has been observed in other studies and reviews that task-specific support changes as a result of behavioural interventions (e.g. PA interventions) but general support does not seem to change in the time frames often studied [61–63].

There were many social wellbeing benefits such as increased social connectivity identified in focus group discussion, but the specific theme of social support was rarely mentioned. It may be that general social support through such community groups may take longer than 1 year to develop. There is evidence that strong group ties are sequentially positively associated between social identification and social support [34], suggesting that the connections formed through the groups may lead increased to social support from group members in the future. This is supported by results from the focus group discussions, where one new member felt she could call on colleagues she met in her new group. Other new members thought it was too soon for this support to be available, but they could see the bonds developing.

### Other social wellbeing changes

In addition to social support and loneliness that were the focus of the quantitative study, the focus group discussions uncovered a number of other benefits of group membership that were related to social wellbeing (see Fig. 3). The social resources theme was of particular interest because it reflected some of the mechanisms that appeared enable social wellbeing changes as a result of being a member of a LAC but were not measured in the survey. The main social resources relating to group membership that were mentioned in the focus groups were social connectedness, development of friendships and opportunity for social responsibility or leadership. As mentioned above, there was wide-spread discussion within the focus groups of the development of social connections through the clubs. Social connectedness is defined as “the sense of belonging and subjective psychological bond that people feel in relation to individuals and groups of others.” ([25], pp1). As well as being an important predecessor of social support, greater social connectedness has been found to be highly important for the health of older adults, especially cognitive and mental health [26, 32, 34, 35, 64]. One suggested theory for this health benefit is that connections developed through groups that we strongly identify with are likely to be important for the development of *social identity* [34], defined by Taifel as: *“knowledge that [we] belong to certain social groups together with some emotional and value significance to [us] of this group membership” (Tajfel, 1972, p. 31 in* [58] *p 2).* These types of groups to which we identify may be a source of “personal security, social companionship, emotional bonding, intellectual stimulation, and collaborative learning and……allow us to achieve goals.” ([58] p2) and an overall sense of self-worth and wellbeing. There was a great deal of discussion relating to the opportunity for social connectedness derived through group membership being particularly pertinent following a significant life event such as moving to a new house or partners becoming unwell or dying and especially retirement. This change in their social circumstance is likely to have triggered the need to renew their social identity by joining a community group. Research with university students has shown that new group identification can assist in transition for university students who have lost their old groups of friends because of starting university [65]. In an example relevant to older adults, maintenance or increase in number of group memberships at the time of retirement reduced mortality risk 8 years later compared to people who reduce their number of group activities in a longitudinal cohort study [66]. This would fit with the original Activity Theory of ageing; whereby better ageing experience is achieved when levels of social participation are maintained, and role replacement occurs when old roles (such as working roles) must be relinquished [67]. These connections therefore appear to assist in maintaining resilience in older adults defined as “the ability to maintain or improve a level of functional ability (a combination of intrinsic physical and mental capacity and environment) in the face of adversity” (p29, [5]). Factors that were mentioned in the focus groups as assisting participants in forming connections with others were shared interest, learning from others, and a fun and accepting environment. It was not possible to assess all life events in the survey study. However, since the discussion from the focus groups suggested this to be an important motivator for joining clubs and potentially a beneficial time for joining them, it would be worth exploring in future studies.

Focus group discussion suggested that an especially valuable time for joining such clubs was around retirement, to assist with maintaining social connectivity. The social groups seem to provide social activity and new roles for these older adults at times of change. It is not necessarily important for all older adults but maybe these ones identify themselves as social beings and therefore this maintenance of social connection helps to continue their social role. Given the suggested importance of social connectivity gained through this organisation, especially at times of significant life events, it would valuable to investigate this further in future and consider encouragement of such through government policy and funding. The majority of these types of clubs exist for older adults in general, but this study emphasises the need for groups such as these to target newly retired individuals specifically and to ensure that they are not seen as ‘only for old people’.

### Strengths and limitations

The use of mixed –methodologies, combining longitudinal survey study analysed quantitatively, with a qualitative exploration through focus group discussions and thematic analysis, was a strength of the current study. It allowed the researchers to not only examine the association between becoming a member of a community group on social support and loneliness over an extended period, but also obtain a deeper understanding of the underlying reasons behind any associations. Given the variability of social support definitions in research [17] and the broad area of social wellbeing, it allowed for open exploration of the topic, to understand associations that may exist but would have otherwise been missed. Embedding the research in an existing community organisation was a strength, although with this also came some difficulties with recruitment. Voluntary coordination of the community groups meant that informing new members about the study was not always feasible or a priority for the volunteers. In addition, calling for new members was innately challenging because they were not yet committed to the club fully. This meant that so some people did not want to commit to a year-long study if they were not sure how long they would be a member of the club. This resulted in slow recruitment and a resulting relatively low sample size and decreased power to show significant statistical differences, which is a limitation of the present study. However, the use of Linear Mixed Models for analysis of the survey data was a strength because it was able to include all data in the analyses and not remove participants if one time point of data was missing, as repeated measures ANOVAs would do. The length of the study (1 year) is another strength, especially compared to previous randomised controlled studies that are typically only 6–16 weeks in length. Drop-out rate in the current study is very low and probably attributable to the benefits of working with long-standing organisations.

The purpose of this study was to explore in detail whether there are any relationships between joining existing community groups for older adults and social wellbeing. The lack of existing evidence in the field meant that a small feasibility-type case study was a good sounding-board for future larger scale research on the topic, despite not being able to answer questions of causality. Owing to the particularistic nature of case studies, it can also be difficult to generalise to other types of organisations or groups unless there is a great deal of similarity between them [68]. There are however, other types of community organisations in existence that have a similar structure to LACVI (Seniors centres [36, 40], Men’s Sheds [38], University of the Third Age [34, 69], Japanese salons [70, 71]) and it may be that the results from this study are transferable to these also. This study adds to the literature around the benefits of joining community organisations that offer social and physical activities for older adults and suggests that this engagement may assist with reducing loneliness and maintaining social connection, especially around the time of retirement.

### Directions for future research

Given that social support trended toward a significant increase, it would be useful to repeat the study on a larger scale in future to confirm this. Either a case study on a similar but larger community group or combining a number of community organisations would enable recruitment of more participants. Such an approach would also assist in assessing the generalisability of our findings to other community groups. Given that discussions around social benefits of group membership in the focus groups was often raised in conjunction with the occurrence of significant life events, it would be beneficial to include a significant life event scale in any future studies in this area. The qualitative results also suggest that it would be useful to investigate whether people who join community groups in early years post retirement gain the same social benefits as those in later stages of retirement. Studies investigating additional health benefits of these community groups such as physical activity, depression and general wellbeing would also be warranted.

## Conclusion

With an ageing population, it is important to investigate ways to enable older adults to age successfully to ensure optimal quality of life and minimisation of health care costs. Social determinants of health such as social support, loneliness and social contact are important contributors to successful ageing through improvements in cognitive health, quality of life, reduction in depression and reduction in mortality. Unfortunately, older adults are at risk of these social factors declining in older age and there is little research investigating how best to tackle this. Community groups offering a range of activities may assist by improving social connectedness and social support and reducing loneliness for older adults. Some factors that may assist with this are activities that encourage sharing interests, learning from others, and are conducted in a fun and accepting environment. Such groups may be particularly important in developing social contacts for newly retired individuals or around other significant life events such as moving or illness of loved ones. In conclusion, ageing policy and strategies should emphasise participation in community groups especially for those recently retired, as they may assist in reducing loneliness and increasing social connections for older adults.

## Abbreviations

FG: Focus group
LAC: Life Activities Club
LACVI: Life Activities Clubs Victoria
LMM: Linear mixed model
PA: Physical activity
WHO: World Health Organisation
